# The effect of vanadium ferrite doping on the bioactivity of mesoporous bioactive glass-ceramics

**DOI:** 10.1039/d2ra04786a

**Published:** 2022-09-08

**Authors:** Sajjad Omidian, Masoumeh Haghbin Nazarpak, Zohreh Bagher, Fathollah Moztarzadeh

**Affiliations:** Faculty of Biomedical Engineering (Center of Excellence), Amirkabir University of Technology (Tehran Polytechnic) Tehran Iran; New Technologies Research Center (NTRC), Amirkabir University of Technology (Tehran Polytechnic) Tehran Iran haghbin@aut.ac.ir; ENT and Head and Neck Research Center and Department, The Five Senses Health Institute, School of Medicine, Iran University of Medical Sciences Tehran Iran; Department of Tissue Engineering & Regenerative Medicine, Faculty of Advanced Technologies in Medicine, Iran University of Medical Sciences Tehran Iran Bagher.z@iums.ac.ir

## Abstract

Bioactive glasses are highly reactive surface materials synthesized by melting or sol–gel techniques. In this study, mesoporous bioactive glass-ceramics doped with different amounts of vanadium and iron ((60−(*x* + *y*)) SiO_2_–36CaO–4P_2_O_5_–*x*V_2_O_5_–*y*Fe_2_O_3_, *x* and *y* between 0, 5 and, 10 mole%) were synthesized using a sol–gel method. Then, their effects on particle morphology and the biomineralization process were examined in simulated body fluid (SBF). N2 adsorption isotherm analysis proved that the samples have a mesoporous structure. In addition, the Fourier-transform infrared spectroscopy (FTIR) spectra of the samples after soaking in SBF for various periods (7, 14, and 21 days) confirmed the presence of new chemical bonds related to the apatite phase, which is in accordance with scanning electron microscopy (SEM) observations. X-ray diffraction (XRD) patterns of the samples after SBF soaking showed that lower amounts of vanadium and iron were associated with the formation of a stable and more crystalline phase of hydroxyapatite. The MTT results showed that the cell viability of mesoporous bioactive glass containing 5% V_2_O_5_ remains more than 90% over 7 days, which indicates the biocompatibility of the samples. To conclude, further studies on these formulations are going to be carried out in future investigations for chemohyperthermia application.

## Abstract 2

Bioactive glasses are highly reactive surface materials synthesized by melting or sol–gel techniques.

## Graphical abstract

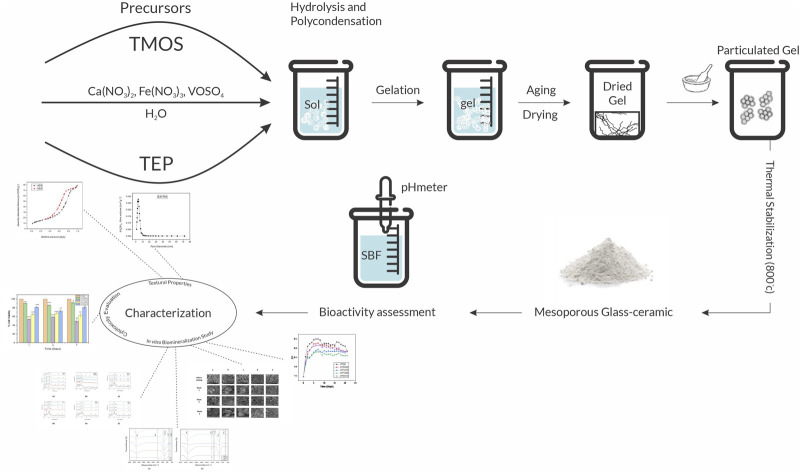

## 1. Introduction

Since the interesting and constructive invention of bioactive glass in 1969 by Prof. Larry Hench, its suitability for various biomedical applications was shown due to its distinctive properties such as antibacterial activity, osteoconductivity, osteogenicity, hemostatic activity, and bioactivity.^1–9^ The ability of bioglass to enhance revascularization, osteoblast adhesion, enzyme activity, and differentiation of mesenchymal stem cells makes it suitable for bone tissue engineering applications.^10–13^ Studies have proved that bioactive glasses possess a higher capability to stimulate bone regeneration in comparison with other bioactive ceramics. In addition, they have shown faster rates of bonding with bones compared with other types of bioceramics in *in vivo* studies. On the other hand, *in vitro* studies have shown that dissolution products of bioglass may stimulate osteoprogenitor cells from the genetic view, which is the cause of their osteogenic properties. Healing of bone defects occurring due to trauma, congenital defects, or disease, *e.g.* osteoporosis or tumor removal can be considered the most important application of bioactive glass ceramics.^1^ A bioactive material is a kind of material that interacts in the body *via* two steps; first, it forms a specific surface reaction, especially with body fluids which develops a hydroxyapatite (HAp)-like layer as a result of interactions within hard and soft tissues during the second step. To maintain the bioactivity of the material in an *in vivo* study, the formation of a HAp-like surface interaction layer after immersing in SBF in an *in vitro* study is fundamental.^10,13–15^

One of the most important properties of bioglass is the ability to develop new properties by modifying its components and manufacturing processes. Besides, glass-ceramics can be produced by applying suitable changes in process variables, which resulted in new capabilities. Whether using melt quenching or sol–gel method of manufacturing, bioglass provides the opportunity to design suitable alternatives for specific applications through tailoring the initial composition or changing the processing conditions. For example, partial crystallization of bioglasses can cause their conversion to glass-ceramics which may enhance their mechanical properties.^16^ On the other hand, glass-ceramics consist of crystalline phases embedded inside an amorphous glassy matrix which may create desired properties.^10^ The appropriate biomedical properties of bioactive glass-ceramics have put them at the center of attention in academic circles in recent decades.^17^ Due to these characteristics, bioactive glass-ceramics are considered more suitable for biomedical applications in comparison to conventional bioglasses.^16^ Also, several commercial products for biomedical applications of bioglass-ceramics were found due to the recent recognition of the field.^17^

There are several methods to synthesize glass-ceramics. Melt quenching and subsequent heat treatment technique can be referred to as the most common way of synthesizing glass-ceramics; however, recently the sol–gel method and solid-state reaction have received more attention.^18^ Sol–gel method notability is due to its chemical homogeneity (molecular level mixing), low processing temperatures, possibility to control size, shape and morphology of the synthesized materials. Moreover, the innate characteristics of the sol–gel technique and the chemical modification of the precursors ease the adjustment of the material properties to a great extent.^19^ There are several factors involved in the sol–gel method synthesis process such as hydrolysis ratio, gelation time, aging, drying and calcination temperature that may tune porous bioactive glasses properties.^20^

On the other hand, magnetic properties are required for some biomedical applications such as hyperthermia and local drug delivery. Some additives may create magnetic properties in bioactive glasses. Such glasses can be crystallized without significantly compromising bioactive properties and can be doped with many elements of the periodic table.^21^ Magnetic nanoparticle systems which display super paramagnetic behaviors tend to exhibit diminutive or no eminence and coercivity whereas showing high amount of saturation magnetization. Therefore, they possess a high potential in biomedicine, magnetic drug delivery, cell-sorting systems, and magnetic refrigeration technology.^22^ In recent years, research on magnetic bioactive glass-ceramics as “thermoseeds” for hyperthermia treatment of cancer, especially deep bone tumors, have received great interests. Commonly temperatures around 42–45 °C can burn and destroy these deep-regional tumors without causing any damages to the normal tissue.^21,23,24^ When implanted around tumors and subjected to the alternating magnetic fields of high frequency, magnetic glass-ceramic particles could produce enough heat by hysteresis and eddy current losses. This magnetic heat generation depends on different variables such as the magnetic properties of the material, the amount of magnetic phase, the strength and frequency of the applied alternating magnetic field, the microstructure and particle size of the ferromagnetic bioactive glass-ceramic.^18,25–27^

In addition to the positive effects of magnetic hyperthermia on cancer treatment, few reports are claiming that magnetic bioactive glass-ceramics may accelerate bone formation.^28^ Commonly, ferromagnetic bioactive glass-ceramics can both generate heat under (externally applied) alternating magnetic fields and have the ability to bond with the living tissues through the formation of a biologically active HAp-like layer; therefore, these materials can be used as thermoseed for hyperthermia and as a substitution for a cancerous-damaged bone. The bioactivity and magnetic properties of these materials have led to extended studies of ferromagnetic bioactive glass-ceramics.^29^

Moreover, researches have shown that vanadium ions stimulate osteoblast differentiation and mineralization in a model of osteoblasts in culture suggesting osteogenic activity of these ions.^30^ Synthesized vanadium ferrite have low coercivity value from the hysteresis loop which means possessing super paramagnetic behaviour.^22^ Vanadium-containing borate glasses have been distinguished based on their high degradation rates in preceding studies. Vanadium therapeutic ions containing materials with high *in vitro* HAp-forming ability tend to show more potentials in bone tissue engineering applications.^31^ The existence of vanadium in the network of the glass plays a central role in the crystallization process; which, in turn has the capability to speed up the reaction of material during immersion in body fluids.^31^

This study aims to synthesize a new vanadium–ferrite containing mesoporous magnetic bioactive glass-ceramics *via* sol–gel method and investigate the effects of magnetic components on structural properties and *in vitro* bioactivity of this bioactive glass-ceramics. The specific innovation of this research is optimizing the amount of vanadium ferrite in the structure of bioactive glass-ceramic and investigating its effects on the structural properties and *in vitro* bioactivity. Also, their magnetic properties will be evaluated and analyzed, especially for the application of chemohyperthermia in the future studies.

## 2. Materials and methods

### 2.1. Synthesis of glass powders

Mesoporous bioactive glass-ceramic (MBGC) powders (60% (SiO_2_ + V_2_O_5_ + Fe_2_O_3_), 36% CaO and 4% P_2_O_5_ (mole%)) were synthesized *via* sol–gel method.^18^ In summary, 2 moles of nitric acid as a catalyst for the hydrolysis reaction and deionized water were mixed in a volume ratio of 1 : 6. The silica precursor tetramethyl orthosilicate (TMOS, Sigma Aldrich) and the phosphate precursor triethyl phosphate (TEP, Sigma Aldrich) were each poured into a water/acid solution and stirred for 1 hour each (ratio of 1 : 12 M TMOS/TEP to water solution). The required amounts of calcium nitrate, iron nitrate and vanadium(ii) sulfate (all of which purchased from Sigma Aldrich) were added to the solution according to the composition of the glass (Table 1) and stirred at 50 °C until all salts were completely dissolved. The resulting sol is kept at 37 °C for 7 days for polycondensation reaction and gel formation. Samples were dried for 24 h at 70 °C and 140 °C in each step. Finally, all samples were thermally stabilized at 800 °C for 2 h at a heating rate of 5 °C min^−1^.

**Table 1:** Chemical composition and codes of different samples (mole percent)

Code	Si_2_O	CaO	P_2_O_5_	Fe_2_O_3_	V_2_O_5_
VF00	60	36	4	0	0
VF0505	50	36	4	5	5
VF1010	40	36	4	10	10
VF1005	45	36	4	5	10
VF0510	45	36	4	10	5

Samples were divided into 5 groups, all of which possess the same mole percent of 36 for CaO and 4 for P_2_O_5_. In VF00 there has been 60 weight percent SiO_2_ without any V or Fe content. In the other samples, the mole percent of SiO_2_ were replaced by addition of 5 to 10 mole percent V_2_O_5_ and Fe_2_O_3_. Table 1 displays the amount of mole percent of each oxides.

### 2.2. X-ray diffraction (XRD)

For XRD analysis, the sintered glasses were grinded and powdered, and analyzed with a Siemens-Brucker D5000 diffractometer which uses Cu-Kα radiation (1.540600 Å and works with voltage and current settings of 40 kV and 40 mA, respectively before and after soaking in SBF. For qualitative analysis, XRD diagrams were recorded in the interval 10° ≤ 2*θ* ≤ 90°at scan speed of 2° min^−1^.

### 2.3. Fourier transform infrared (FTIR) spectroscopy

Samples of MBGC powder were investigated by FTIR Spectroscopy of the Bomem MB 100 spectrometer. For IR analysis, at first 1 mg of powdered sample was thoroughly mixed with 300 mg of KBr (infrared grade) and palletized under vacuum. Then the pellets were analyzed in the range of 400–4000 cm^−1^ at the scan speed of 23 scan per min with 4 cm^−1^ resolution.

### 2.4. Particles size and particle size distribution

The size distribution of MBGC particles in aqueous dispersions was determined by dynamic light scattering. Measurement was performed at 25 °C with a detection angle of 90° using a particle size analyzer (Zetasizer nano ZS, Malvern Instruments Ltd., UK).

### 2.5. Brunauer–Emmett–Teller (BET)

Surface area and pore size distribution were measured by the BET method using a surface area and porosimetry analyzer (Micromeritics ASAP 2020). The samples were degassed in vacuum at 150 °C for 18–20 h for the removal of moisture from the pores and analyzed with nitrogen adsorption.

### 2.6. Scanning electron microscope (SEM)

The microstructure of the samples was evaluated using SEM. After the sample was covered with a thin layer of gold (Au) by sputtering (EMITECH K450X, England), the morphology was observed in a scanning electron microscope (SEMPhilips XL30) operated at an accelerating voltage of 15 kV.

Also, an Energy Dispersive X-ray analyzer (EDX) (MIRA 3 TESCAN, USA) connected to SEM was used to investigate pseudo quantitatively chemical compositions.

### 2.7. Dynamic light scattering (DLS)

The particle size distribution of the BGCs in water dispersion was determined by dynamic light scattering using a particle size analyzer (Zetasizer Nano ZS, Malvern Instruments Ltd., U. K.) at 25 °C with a 90° detection angle.

### 2.8. Preparation of SBF solution

The SBF solution was prepared according to Kokubo's specifications.^32^ Some reagents such as NaCl, NaHCO_3_, KCl, K_2_HPO_4_·3H_2_O, MgCl_2_·6H_2_O, CaCl_2_, trishydroxymethyl aminomethane [Tris buffer, (CH_2_OH)_3_CNH_2_], and 1 N HCl, were purchased from Merck Inc. The SBF solution was prepared by dissolving NaCl, KCl, NaHCO_3_, MgCl_2_·6H_2_O, CaCl_2_ and KH_2_PO_4_ (analytical grade) in distilled water and buffered at pH = 7.25 with Tris buffer and HCl 1 N at 37 °C.^33^

### 2.9. Biological evaluation

#### 2.9.1. *In vitro* biomineralization study

The SBF immersion test is a fast, simple and inexpensive method, commonly used for biomineralization studies of bioactive materials which is an accurate simulation of the osteogenic conditions of bone tissue, and is a valid method to study the biological activity of the material and the mechanism of bone bonding.^34^*In vitro* study was performed by immersing the sample of MBGC powder in a solution of 1.5 mg of powder per milliliter of fluid at SBF in static condition at 37 °C for 7, 14 and 21 days. After this time, the powder was separated from the solution and dried in an incubator. The ion concentrations of released silicon (Si), calcium (Ca), phosphorous (P), iron (Fe), and vanadium (V) were measured after immersing the MBGC powder in SBF with an inductively coupled plasma-optical emission spectrometer (ICP-AES 730-ES Varian USA). In addition, the pH of the SBF solution was measured with a calibrated pH-meter at each step using a Corning 320 pH-meter. In addition, the surface of the samples was analyzed by XRD and SEM before and after SBF immersion.

#### 2.9.2. Cytotoxicity evaluation

The colorimetric assay developed by Mosmann^35^ and modified by Edmondson *et al.*^36^ was used as a test for L929 cell proliferation and survival in this study. A 3-(4,5 dimethylthiazol-2-yl)-2,5-diphenyl tetrazolium bromide dye solution (MTT) (Sigma, St Louis, MO) was prepared as 0.5 mg mL^−1^ in PBS at 37 °C just before use. A total of 20 μL MTT dye was added to each well and incubated at 37 °C in air containing 5% CO_2_ and at 95% relative humidity for 4 hours under dark conditions. After incubation, the MTT was aspirated and the formazan product was solubilized in 50 μL dimethyl sulfoxide (DMSO) (Sigma-Aldrich, St Louis, MO). The plates were shaken before the optical densities were measured at 570 nm, using an enzyme-linked immunosorbent assay (ELISA) plate reader (Dynatech-MRX; Dynatech Laboratories Inc., Alexandria, VA, USA). All assays were repeated thrice to ensure reproducibility. The absorption value obtained with the control was deemed to indicate 100% viability. The percentage of viable cells was determined using the following formula:

1 Percentage of viable cells = (*A*/*B*) × 100,

Where, *A* = viable cells in the experimental well and *B* = viable cells in the control well. More than 90% cell viability was considered as noncytotoxic, 60% to 90% as slightly cytotoxic, 30% to 59% as moderately cytotoxic, and less than 30% cell viability was considered as strongly cytotoxic.^37^ In this study, MBGC without any vanadium or iron component (VF00) was taken as the control sample.

#### 2.9.3. Statistical analysis

All experiments were performed in the triple replicates. The results are given as the mean standard error (SE). Statistical analysis was performed using one-way ANOVA and Tukey test. A *P* value less than 0.05 was considered significant.

## 3. Result & discussion

### 3.1. SEM observation & obtaining size distribution of the synthesized MBGC

**Fig. 1:**
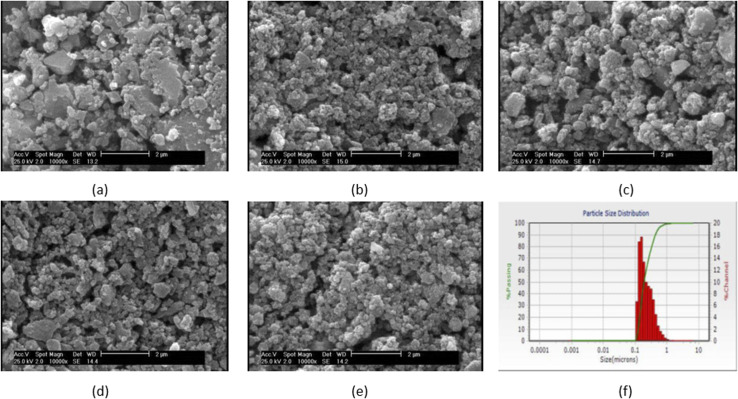
SEM micrograph of the synthesized particles (a) VF00 (b) VF0505 (c) VF1010 (d) VF1005 (e) VF0510 and (f) particle's size distribution of VF1010 particles.

Fig. 1(a–e) shows the SEM micrograph of synthesized samples. As can be seen, before immersion, heterogeneous surfaces consisting of randomly sized particles with sharp edges and gaps between them are formed as the results of similar research in this field.^18,38^ Also, as shown in this figure, the particle size ranges from 100 to 200 nm. Since the particle's size distribution of samples were approximately similar, only one of the five samples is shown in Fig. 1(f) which the average diameter of VF1010 particles is 191 nm. The results of DLS analysis for the size of the synthesized particles are presented in Table 2 (*n* = 3).

**Table 2:** Average particle size of different synthesized particles

Sample code	Average size (nm)
VF00	205 ± 40.5
VF0505	180 ± 25.5
VF1010	191 ± 73.7
VF1005	172 ± 20
VF0510	218 ± 22.5

### 3.2. Textural properties

**Fig. 2:**
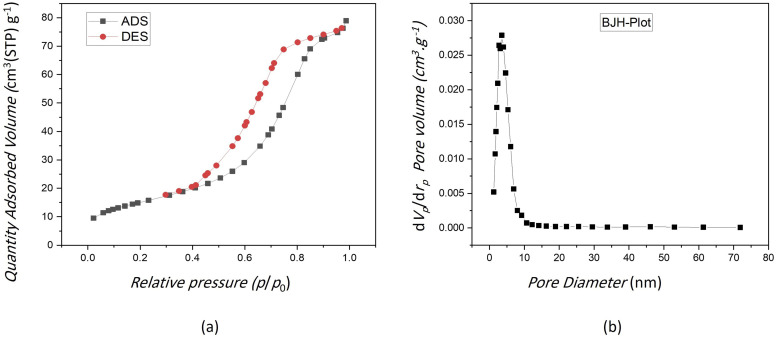
(a) N_2_ adsorption–desorption isotherms of VF00 sample. (b) Mesopore size distribution of VF00 sample.

The results of study performed by Li *et al.* indicated that the mesoporous bioactive glass has a larger surface area and higher pore volume compared with the non-mesoporous bioactive glass.^39^Fig. 2(a) displays the N2 adsorption–desorption isotherm pattern for the VF00 is of type IV for the BET analysis. Parameters corresponding to the pore characteristics of all samples were measured. Table 3 indicates average pore size, BET surface area and total pore volume of the samples. Fig. 2(a) shows a hysteresis loop for VF00 sample and displays the mesoporous structure of the sample to induce capillary condensation in order to form a hysteresis loop. This proves the existence of mesoporous structures in those samples. Fig. 2(b) displays the pore size distribution of sample VF00 indicating that bioglass have pore diameters of around 5–50 nm which based on International Union of Pure and Applied Chemistry's (IUPAC) nomenclature, mesoporous materials have structure diameters ranging from 2 to 50 nm with some exclusive outstanding properties.^40^

**Table 3:** Average pore size BET surface area and total pore volume of glass-ceramics

Sample codes	VF00	VF0505	VF1010	VF1005	VF0510
Average pore size (nm)	9.0245	10.886	18.434	17.276	19.005
Total pore volume (cm^3^ g^−1^)	0.1221	0.058584	0.169	0.2229	0.090286
BET surface area (m^2^ g^−1^)	54.121	21.526	36.661	51.615	19.003

A more suitable design for mesoporous silica-based structure with appropriate pore structures, pore sizes, and surface modification has provided more efficient drug carriers.^41^ The results in Table 3 showed that specific surface area and total pore volume of VF00 sample were more than other samples; however, VF00 does not have vanadium ferrite; therefore VF1005 shows better parameters among the samples containing vanadium ferrite. This indicates that more drugs can be loaded into these nanoparticles because the larger the total pore volume and the specific surface area, the greater the capacity to carry more cargo molecules.^42^ This helps us to select the best sample having relatively high potential for drug delivery applications. Since the average pore size of VF0510 is more than other samples, it is a suitable candidate for drug delivery. It is known that the adsorption of proteins (such as albumin and fibronectin) or drugs are increased as the nanopore size increases.^43^ Thus, in a way VF00 shows better cargo molecules carrier characteristics due to its specific surface area and the total pore volume.^44^ Jia *et al.* have found that the larger the pore size, the higher is the drug loading capacity, the faster the release rate, the higher is the *in vitro* anticancer activity.^45^ However, VF0510 can be better suited for drug delivery purposes according to its higher average pore size, which is considered for the future drug release studies.

### 3.3. Bioactivity assessment

#### 3.3.1. pH changes in SBF composition and surface morphology evaluations

**Fig. 3:**
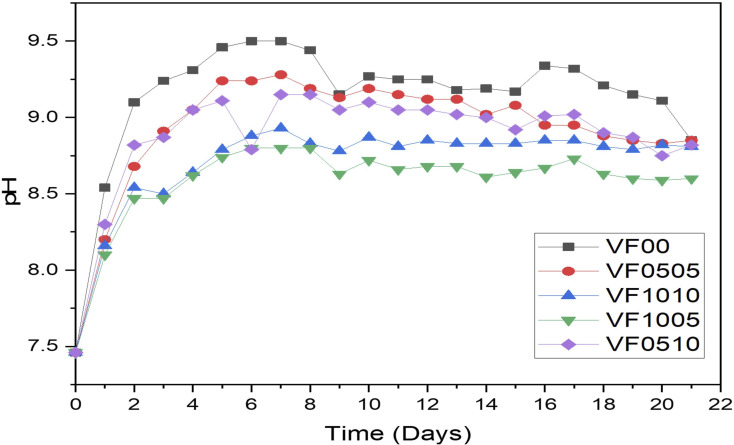
pH variation of MBGCs during 21 days soaking in SBF.

pH measurements were carried out on the SBF solution for 21 days in order to check the hydrolytical stability of MBGCs. The degradation of MBGCs is usually accompanied by the dissolution of soluble species and ions that depend on the composition of the glass.^18^ The data obtained from the pH measurement of MBGCs soaked in SBF at 21 days are shown in Fig. 3 pH changes show a noticeable increase from 7.46 to 9.5, 9.28, 8.93, 8.83, and 9.15, respectively, after soaking for 7 days. This pH rising continued to amounts 9.19, 9.02, 8.83, 8.61 and 9, respectively after 14 days soaking in SBF. However, the pH variations changed to 8.85, 8.85, 8.81, 8.6 and 8.82, respectively after 21 days of soaking. pH variations of bioactive glass-ceramics indicates a significant change during the immersing time (Fig. 3). Moreover, mesoporous bioactive glass VF00 showed the largest increase in pH among all samples. This increase in pH is mainly due to the exchange of Ca^2+^ ions of the MBGC with H_3_O^+^ ions in the SBF solution.^46^ In other words, leaching of ions from the glass matrix increases the silicon and calcium concentrations in SBF and subsequent calcium phosphate formation takes place, reducing the phosphorus and calcium concentrations in solution.^47^ Therefore, before the formation of hydroxyapatite, a layer of silica gel is formed on the surface of the glass. From Fig. 3, it is clear that the pH increased very rapidly after the first 4 days of immersion in SBF for all MBGC samples. The pH trends is as follows VF00 > VF0505 > VF0510 > VF1010 > VF1005. After 7 days, the pH amounts of all samples reached the maximum level. Thereafter, a slight decrease in the pH value was observed for up to 10 days and then remains constant up to 14 days. After this period of time, the pH slowly increases.

As mentioned above, the change in pH is mainly due to ion leaching. Hence, the increase in pH of the SBF solution indicates a decrease in the concentration of H^+^ ions due to the substitution of cations in the MBGC. A relative decrease in the pH of the SBF solution after 9 days was also observed, which could either lead to silanol formation or to breaking of the glass network.^47,48^ The morphological properties of the MBGCs also indicate the formation of an apatite-like layer on the surface of the samples following immersion in SBF.

Fig. 3 shows pH variation of MBGCs during 21 days soaking in SBF. As can be seen V_2_O_5_ addition to the glass samples delays their bioactivity. This is the result of the comparison of the rate of increase in pH between samples VF0505 and VF1005. On the other hand, adding Fe_2_O_3_ increases the bioactivity of the glass sample compared to the sample containing vanadium. This is obvious in the VF1010 and the VF1005 samples.

**Fig. 4:**
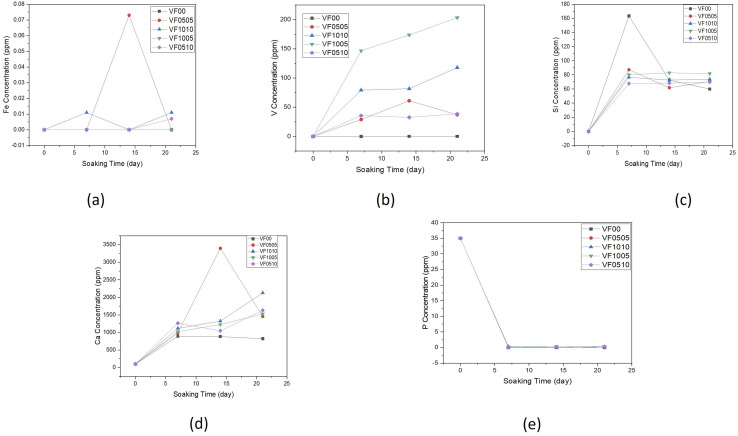
Variation of elemental concentration in the SBF with soaking time for (a) iron, (b) vanadium, (c) silicon, (d) calcium and (e) phosphorus.

Fig. 4(a–e) shows the changes in concentrations of Si, Ca, V, P and Fe in SBF solution during 0, 7, 14 and 21 days, as measured by ICP-AES. It can be seen that when MBGC reacts with SBF, both chemical and structural changes occur over the time on the MBGC surface, and the accumulation of dissolution products alter both the chemical composition and pH of the solution. Concentrations of Ca and Si, after reaching a maximum at day 7, gradually decreased up to 21 days with the exception of VF0505, where Ca concentrations peaked at 14 days and Ca concentrations of VF1010 and VF1005 increased slightly until 21 days. These changes in the composition of SBF depend on the five chemical steps in the formation of hydroxyapatite, hydroxylapatite carbonate and calcium carbonate on the surface of MBGC nanoparticles: glass dissolution and ionic exchange between the glass surface and the biological medium, repolymerization of a silica rich layer on the surface, formation of an amorphous calcium phosphate layer and finally crystallization of this layer into a carbonated hydroxyapatite, which Hench *et al.* have described before.^49^ These results are also consistent with the pH measurements of MBGC in SBF. Fe concentration in SBF solution was less than 0.073, 0.011 and 0.007 ppm after 21 days for VF0505, VF1010 and VF0510, respectively. In addition, V concentration in the SBF solution was below 37 and 38 ppm after 21 days for VF0505 and VF0510, respectively. These amounts are almost negligible and released ions are unlikely to cause serious biocompatibility problems *in vitro* and *in vivo.*^18,50,51^ However, V concentration raised for VF1010 and VF1005 up to 117 and 203 ppm, respectively which could be a challenge for biocompatibility of them because of the possible toxicity of high amounts of vanadium.^52^

**Fig. 5:**
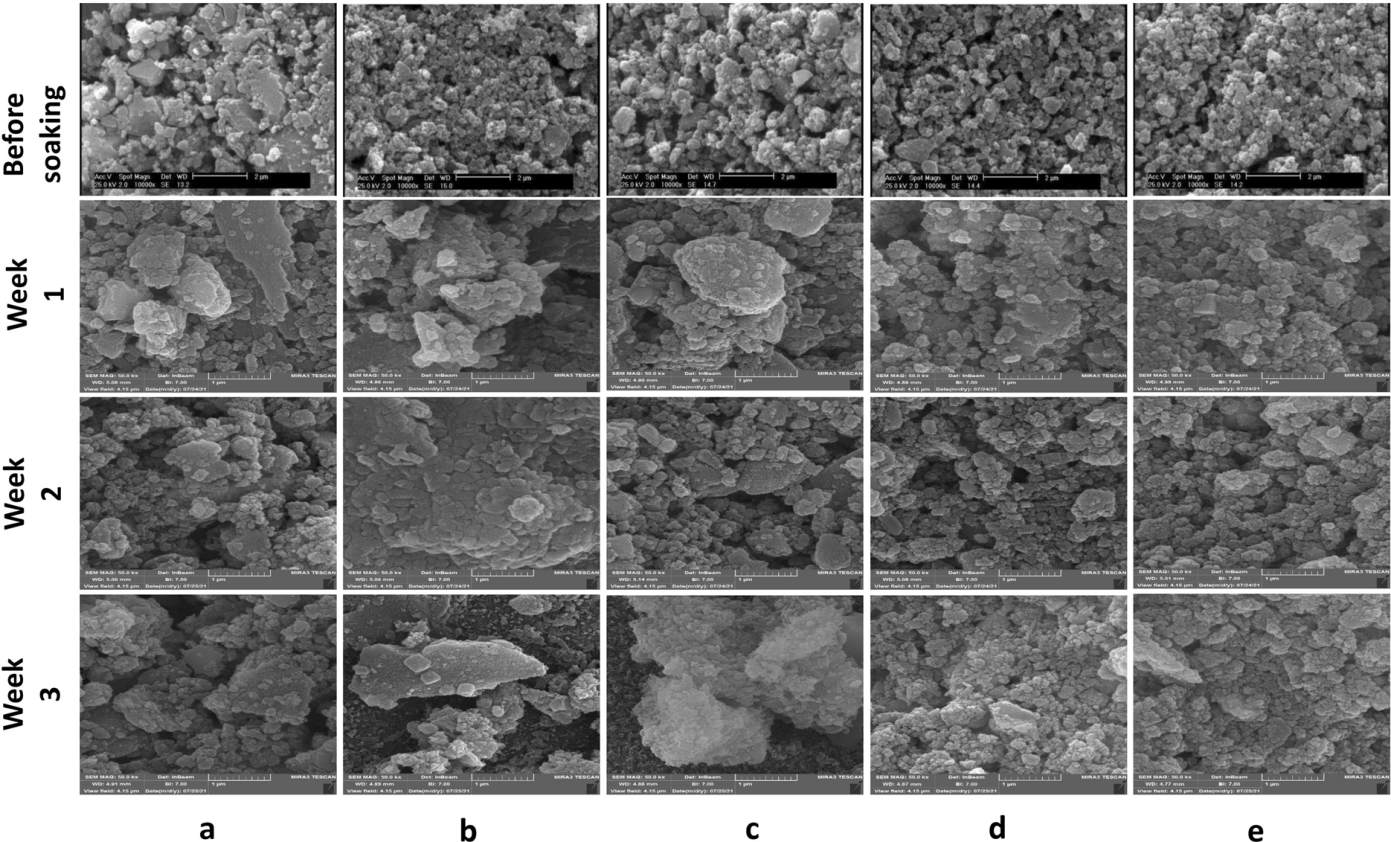
SEM micrographs of glass-ceramic samples before soaking and after soaking in SBF for 1, 2, and 3 weeks. (a) VF00, (b) VF0505, (c) VF1010, (d) VF1005 and (e) VF0510.

Next, MBGCs were soaked in SBF solution for 21 days. Then, the samples were removed and filtered and morphological studies were performed to examine the formation of hydroxyapatite on their surface at different time intervals. The surface morphology of all samples after 7 days in SBF is shown in row 2 Fig. 5(a–e) as can be seen with increasing the V_2_O_5_ content from 5% to 10% (VF1005 *vs.* VF0505), the apatite layer formation is decreased. In addition, the soaking time also directly affects the formation of the MBGC surface layer. Although ferric glasses have increased vanadium ferrite containing (VF1010, VF1005 and VF0510), they show relatively less apatite layer formation after immersion in SBF solution. This may be due to the higher magnetic properties of glass-ceramics and therefore result in the reduction of bioactivity.^18^ XRD analysis of MBGCs also confirmed the formation of crystalline phases. Less apatite layer formation after immersion in SBF may be due to the coulsonite crystalline phase observed in these glasses that reduced the bioactive properties of the glass-ceramic, and the effects of the vanadium phase on the bioactivity. In addition, the pH value is lower for VF1010 and VF1005 glass-ceramics, which indicates their slower dissolution rate. However, after 14 and 21 days of immersion in SBF, all MBGC samples showed the formation of flakey Hap-like particles on their surface.

The variation of shape as well as the size of the hydroxyapatite particles are directly dependent on the pH of the reaction medium^53^ and as displayed in Fig. 3, the pH of different samples are markedly different, which has affected the shape and size of the hydroxyapatite formed on the surface of the samples. Hydroxyapatite can form in a variety of morphological shapes, including sphere, rod, needle, fiber, whiskers, prism, plate, flake, sheet, bundles of rods or needles or fibers, flower, clusters of nanotubes, oriented bundle, and porous microsphere or mesoporous sphere.^54^ It can be said that the preferred structure of crystalline hydroxyapatite in biomedical applications is a flake-like structure;^55^ therefore, as can be seen all samples showed relatively suitable structure for biomedical application after 21 days immersion in SBF.

It can be considered from surface micrograph that the surfaces of all sample are covered with certain types of nanoparticles after 7 days. Row 2 Fig. 5(b) indicates that the formation and crystallization of apatite layer were favorable for the VF0505 sample, which is consistent with the obvious presence of the HAp-like phase.

After soaking in SBF for 14 days, coverage became much more apparent for all samples but with obvious morphological differences. However, such microstructural features are much less distinct on the surfaces of VF00 and VF0505. SEM morphological analyzes after bioactivity assay in SBF (rows 3 Fig. 5) showed excellent apatite-forming ability of all samples. Spherical calcium phosphate agglomeration was observed on the surface of all samples following 7 days SBF immersion, confirming the bioactivity of the samples. After seven days immersion in SBF, apatite-like structures also formed on the surface of glass-ceramics. After two weeks immersion in SBF, a calcium phosphate coating formed from nanostructured spherical aggregates, the morphology of closely resembled hydroxyapatite, was observed on the surface of all samples.

In addition, as can be seen in Fig. 3, the pH changes of the VF1010 and VF1005 glass-ceramics is low, indicating a slow dissolution rate. After 21 days soaking in SBF, all MBGC samples showed the formation of flaky-like particles on the surface (row 4 Fig. 5(a–e)).

#### 3.3.2. FTIR analysis of MBGC before and after immersion in SBF

**Fig. 6:**
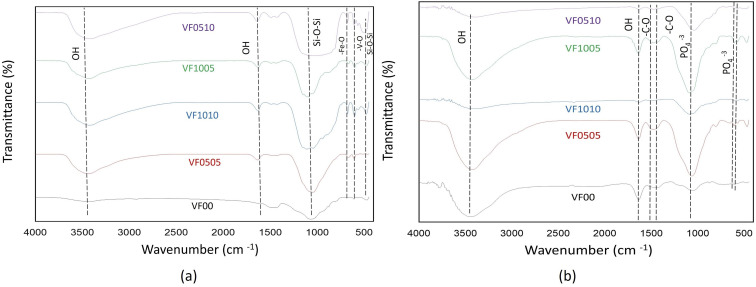
FTIR spectra of MBGC samples (a) before soaking in SBF (b) after soaking in SBF for 14 days.

Fig. 6 shows the FTIR spectra of the samples before and after immersion in SBF up to 14 days. Similar to other SiO_2_-based bioactive glasses that contain SiO_2_ as the main component, Fig. 6(a) shows the main characteristic of a tetrahedral SiO_4_ silicate lattice in the range 400 to 500 cm^−1^, referring to the bending mode of the oscillatory Si–O–Si. The metal–oxygen (Mtetra–O) vibrational mode in the range 500 to 600 cm^−1^ is evidence of the formation of a spinel phase in this material. Metal oxide bonding (M–O–M) confirms the formation of vanadium ferrite. The peaks in the range 860–940 cm^−1^ and 1000–1100 cm^−1^ are attributed to Si–O and Si–O–Si stretching, respectively. The broad band at about 3419 cm^−1^ is related to the presence of (hydroxyl) O–H groups on the surface. The absorption bands at around 3400 and 1630 cm^−1^ are assigned to the hydroxide. Fig. 6(b) shows the FTIR spectrum of MBGC after 14 days immersion in SBF. The transmission spectrum band of MBGC confirms the formation of calcium phosphate compound (HAp-like layer). The doublets at 603 and 565 cm^−1^ are typical of calcium phosphate compounds. The binding of calcium phosphate and carbonate is important for characterizing calcium phosphate compounds. Phosphate groups have four modes that are active in the infrared region: bending vibration of PO_4_ at 560–610 and 430–460 cm^−1^, asymmetric stretch broad band at 1000–1150 and 960 cm^−1^. It should be noted that carbonated HAp also has a characteristic peak in carbonate binding. Peaks in the range of 1421–1631 cm^−1^ corresponded to C–O oscillations, and the wide band around 3418 cm^−1^ was related to the presence of (hydroxyl) OH groups on the surface. Absorption bands of 3400 and 1630 cm^−1^ are assigned to hydroxyl groups.

**Fig. 7:**
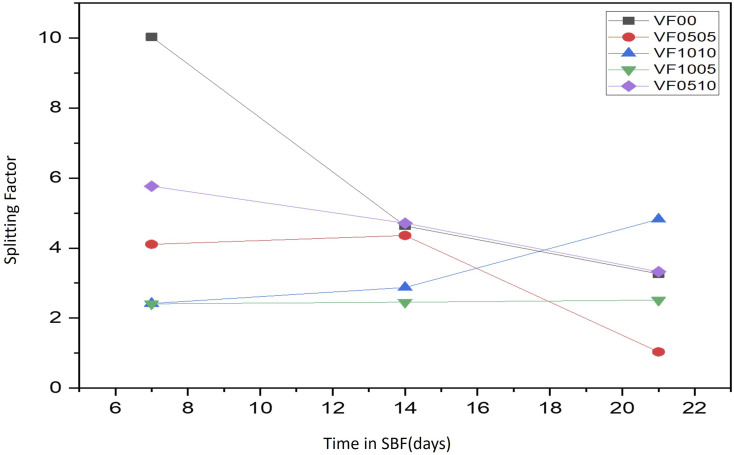
SF values for all glass samples after soaking in SBF for 7, 14 and 21 days.

As already mentioned, FTIR spectroscopy has a characteristic peak in the spectral range of 500–620 cm^−1^, which belongs to the bending mode of PO_4_ oscillations. These characteristic peaks consist of two peaks that correspond to optical horizontal and vertical frequencies. However, amorphous calcium phosphate provides a wide single absorption band in this region due to the structural distortion of the lattice. It should be noted that the double peaks of the phosphate bending frequency near 565 and 603 cm^−1^ are increasingly separated or cleaved with increasing crystallinity.^56,57^ As HAp becomes more crystalline, the hexagonal structure becomes apparent. As a result, PO_4_ oscillations are clearly defined in this region and doublet peaks are observed. Therefore, this division of the bending mode of PO_4_ oscillations in the conversion of HAp from amorphous to crystalline phase may be a crystallinity index.^56^ The splitting factor (SF) was obtained from the FTIR spectrum by normalizing the sum of the absorbances at 565 and 603 cm^−1^ from the bent bond of the PO_4_ bond to the minimum between the two peaks. The crystallinity of the particles was determined from the split coefficients obtained by normalizing the sum of the absorbance at 565 and 603 cm^−1^ to the minimum between the doublets by Weiner and Bar-Yosef.^58^ Finally, the splitting factor is known to quantify the degree of splitting of PO_4_ bond bending peaks (565 and 603 cm^−1^) and increase with increasing crystallinity.^59,60^ Currently, the proposed splitting factor is the major degree of crystallinity by adding the peak heights of 565 and 603 cm ^−1^ and dividing that value by the height of the valley between them. Fig. 7 shows the SF values for all glass samples after being soaked in SBF for 7, 14, and 21 days. As expected, the samples with high vanadium content (VF1005 and VF1010) have lower SF values, so the crystallinity of the HAp-like layer formed on this glass surface is lower than the other samples. On the other hand, the SF value of HAp was shown to be about 5.8.^57^ As shown in Fig. 7, the SF of VF00 is the highest, followed by VF0510. Therefore, the hydroxyapatite-like layer of VF0510 has a higher crystallinity than other MBGCs. Therefore, among the samples containing vanadium ferrite, VF0510 has the most abundant and closest SF value to hydroxyapatite.

#### 3.3.3. XRD analysis of MBGC before and after immersion in SBF

**Fig. 8:**
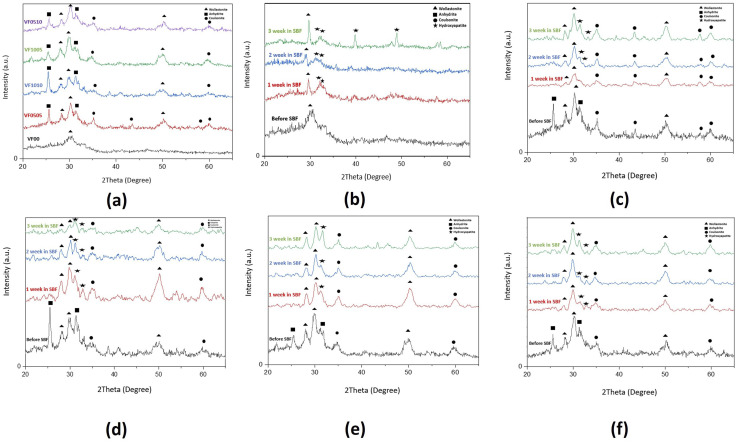
XRD analysis of bioactive glass-ceramics before and after soaking in SBF for 7, 14 and 21 days: (a) before soaking, (b) VF00, (c) VF0505, (d) VF1010, (e) VF1005 and (f) VF0510; ▲: wollastonite, ■: anhydrite, ●: coulsonite, ★: hydroxyapatite.

Fig. 8 shows the XRD pattern of each sample with different immersion times in SBF. It can be seen in Fig. 8(a) that wollastonite structure (CaSiO_3_) is formed on the glass-ceramics. It is shown that all sintered powders, especially VF00 sample, have amorphous phase coexist with some crystal phases Fig. 8(a). The two close XRD peaks at about 28.8 and 30.01 degrees are the main characteristic peaks of wollastonite with the (JCPDS 42-0547) card number.^57^ Peaks at around 49.83 degrees can also be attributed to wollastonite. In the XRD pattern of samples containing vanadium before exposure to SBF, due to the presence of vanadium sulfate as a vanadium precursor and high calcium content (about 12 grams of calcium nitrate as a calcium precursor in all samples), the characteristic of calcium sulfate in form of anhydrite (CaSO_4_) peaks with (JCPDS 072-0916) card number^61^ is shown. Due to the high absorption and degradation rate of calcium sulfate,^62,63^ exposure to SBF after 7 days no longer shows any trace of calcium sulfate. Calcium sulfate exhibits biocompatibility and biodegradability, as well as an excellent capability for bone fusion.^64,65^ It should be noted that previous studies have proven the positive effect of the presence of calcium sulfate next to bioactive glass.^63^ The characteristic peak at about 35 degrees can be attributed to the main coulsonite (FeV_2_O_4_) peak with the (JCPDS 01-075-0317) card number^66^ which is displayed in (Fig. 8(c–f)). VF0505 shows the characteristic peaks of coulsonite in a relatively larger scale compared to the other samples (Fig. 8(c)). As previously stated in SEM and FTIR analysis and pH changes of samples after SBF exposure, due to the bioactivity of the samples, the hydroxyapatite structure is formed on the surface of the samples. The characteristic peaks of hydroxyapatite (Ca_10_(PO_4_)_6_(OH)_2_) in 2 theta angles between 31 and 33 degrees with the (JCPDS 00-009-0432) card number^54^ can be seen in Fig. 8. It is clear that the sample without vanadium and ferrite VF00 (Fig. 8(a)) has shown a higher bioactivity and more crystallinity of the hydroxyapatite layer formed on the bioglass. The XRD patterns of the VF00 sample showed most of the characteristic peaks of hydroxyapatite. While the main characteristic peaks of hydroxyapatite in VF0505 became sharper over the time; in the next three samples, VF1010, VF1005, and VF0510 the characteristic peaks of hydroxyapatite became weaker Fig. 8(d–f).

**Fig. 9:**
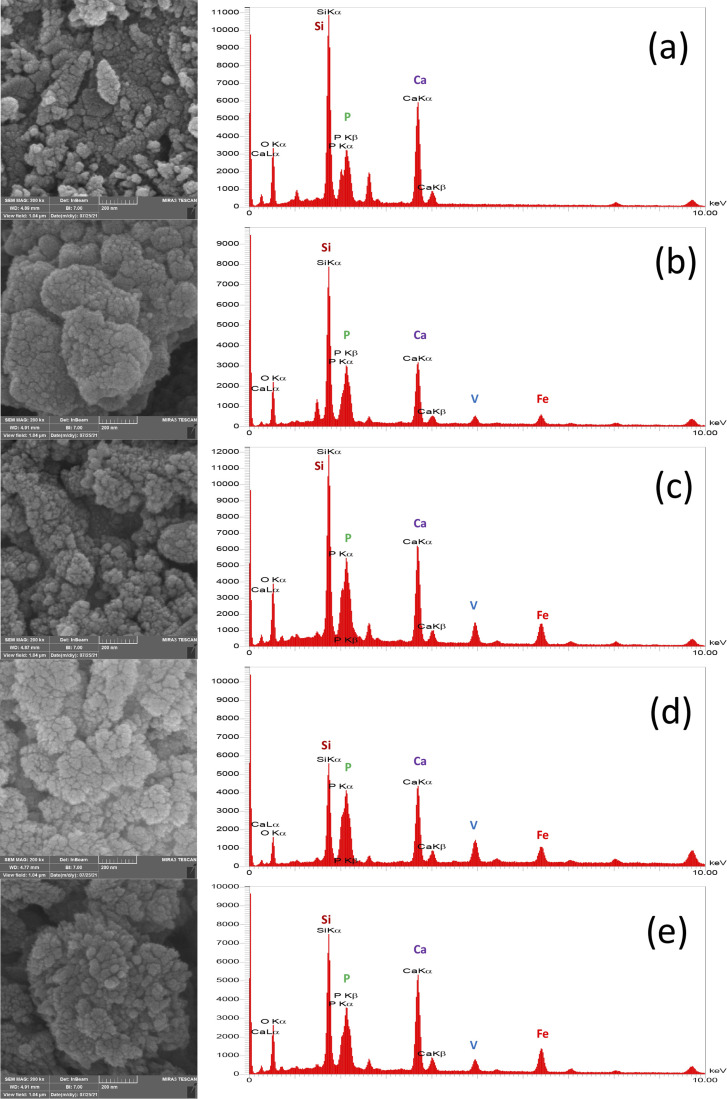
Apatite layer formation and their corresponding EDX curves for (a) VF00 (b) VF0505 and (c) VF1010 (d) VF1005 (e) VF0510 MBGCs after 21 days soaking in SBF.

As can be seen in the micrographs, the entire MBGC surfaces are covered by a layer of calcium phosphate, which creates a flaky-like apatite coating. According to what can be seen in the observations, the addition of vanadium and iron did not cause the loss of bioactivity and the resulting changes were not significant. Also, according to what was said, HAp-like phases are formed on the surface of all samples. Fig. 9 shows the results of EDX analysis after 21 days of immersing in SBF. The EDX spectrum shows all the characteristics of the glass.^18^ Also, as can be seen in Fig. 9(a), which does not contain iron and vanadium (VF00), the characteristic peaks of these two elements are not seen, but in Fig. 9(b), which is for VF0505, the characteristic peaks of vanadium are seen at 4.97 keV and iron at 6.42 keV. For VF1010, which contains more iron and vanadium, the intensity of the peak increases (Fig. 9(c)). Also, for VF1005 in Fig. 9(d), the peak of vanadium is more intense compared to iron, and in VF0510 (Fig. 9(e)), the opposite is true, the peak of iron is more intense compared to vanadium; This results can be a validation of the synthesis correctness. The ratio of calcium to phosphorus (Ca/P) for samples is 2.3, 1.77, 1.77, 1.34 and 2.3, respectively. The Ca/P ratio of stoichiometric hydroxycarbonate apatite is equal to 1.67. Therefore, the Ca/P ratio related to VF00 and VF0510 samples indicate the formation of non-stoichiometric biological apatite. The Ca/P ratio related to VF0505 and VF1010 samples indicate that the Ca/P ratio of these two samples is close to the ratio of hydroxycarbonate apatite. Also, the Ca/P ratio of VF1005 sample is in the range of octacalcium phosphate.^67^ And this result is consistent with the results obtained from the pH observations shown in Fig. 3 and also the amount of SF shown in Fig. 5, and assigns the lowest amount of bioactivity to VF1005 sample. Therefore, according to all the topics raised, we can conclude that for bioactivity, this order exists in the samples: VF00 > VF0510 > VF0505 > VF1010 > VF1005.

### 3.4. Cytotoxicity evaluation

**Fig. 10:**
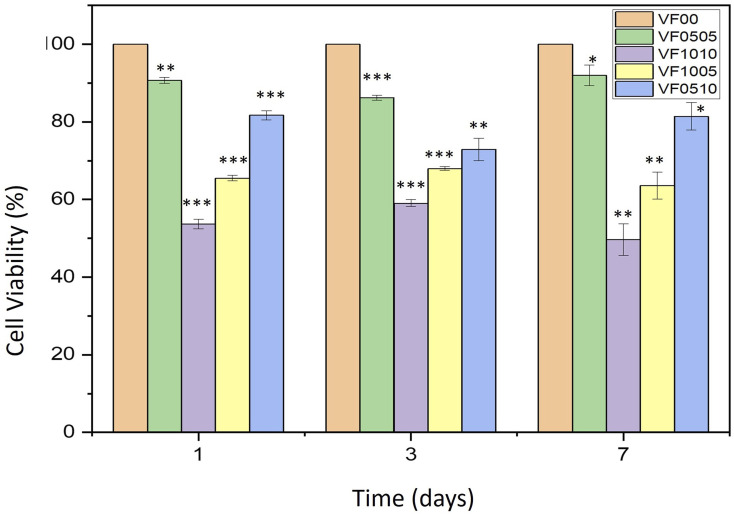
Proliferation of L929 fibroblasts cultured on VF0505, VF1010, VF1005, VF0510 and VF00 as a control. *P ≤ 0.05; **P ≤ 0.01; ***P ≤ 0.001 (difference compared with the control sample).

Cells that come into contact with the surface of biomaterials must attach and spread before they can grow. The quality of this adhesion affects their morphology and their ability to proliferate and differentiate.^68–70^Fig. 10 shows the cell viability of the L929 fibroblasts on VF0505, VF1010, VF1005 and VF0510 thermoseeds after 1, 3 and 7 days. The values of cell viability (%) is displayed in the Table 4. The results display expected slower cell growth on these materials than on a VF00 which was used as the control. Furthermore, differences were found in VF1010 and VF1005 after 1, 3 and 7 days. This delay seems to be related to the composition of the MBGC. It should be noted that other studies also observed that the high percentage of vanadium and iron in the composition led to lower cell growth.^18,71^ The VF0505 and VF0510 samples show a relative decrease in cell viability at 3 days and a relative increase at 7 days. This is probably due to the vanadium and iron contents compared to the control sample (VF00), which may cause relative cytotoxicity (the initial effect of V and Fe) at 3 days. After that, these samples may have slowly compensated for lag and compromised by allowing time to elapse compared to control samples in 7 days. On the other hand, the VF1010 and VF1005 samples show a relative increase in cell viability after 3 days and a relative decrease after 7 days. It might be the high vanadium-containing effect that took longer to show a toxic effect compared to the control sample, and this reduction was observed during day 3 to day 7 of this study. All samples showed significant differences compared to the control group (*p*-values ≤ 0.05). After 7 days VF0505 and VF0510 samples shows no cytotoxic behavior; on the other hand, VF1005 sample shows slightly cytotoxic behavior and VF1010 sample shows moderately cytotoxic behavior, as expected. This shows the cell viability is affected by the percentage of vanadium and iron in the composition. Therefore, samples VF0505 and VF0510 display higher cell viability, as a measure of biocompatibility, and may be better candidates for biomedical application.

**Table 4:** Cell viability (%) of samples, with VF00 control, after 1, 3 and 7 days in MTT assay (*n* = 3)

Sample code	Cell viability (%) after 1 day	Cell viability (%) after 3 days	Cell viability (%) after 7 days
VF0505	90.69 ± 0.76	86.24 ± 0.66	92.02 ± 2.61
VF1010	53.73 ± 1.25	59.05 ± 0.88	49.65 ± 4.06
VF1005	65.51 ± 0.68	67.98 ± 0.47	63.6 ± 3.51
VF0510	81.72 ± 1.15	72.93 ± 2.89	81.42 ± 3.58

## 4. Conclusions

Mesoporous bioactive glass-ceramics doped with vanadium and iron were synthetized using a sol–gel method. Primary characterization showed that the glass-ceramics had a spherical form and a pore distribution in the meso range (2–50 nm). In vanadium–ferrite containing MBGCs, the VF0510 and VF0505 samples generally showed more suitable properties considering all aspects of choosing an optimal formulation for the hyperthermia application that refers to their chemical composition and textural characteristics. VF0510 can be considered the final and more suitable result for this study and also for the future investigations in chemohyperthermia application due to its higher vanadium ferrite content compared to VF0505. In conclusion, VF0510 (45SiO_2_–36CaO–10Fe_2_O_3_–5V_2_O_5_–4P_2_O_5_) is chosen as the best compromise between bioactivity, biocompatibility and probable magnetic properties. Therefore, further studies on this formulation are going to carry out in future investigations.

## Author contributions

Sajjad Omidian: conceptualization, resources, investigation, formal analysis, visualization, writing–original draft. Masoumeh Haghbin Nazarpak: conceptualization, funding acquisition, resources, validation, writing–review and editing. Zohreh Bagher: resources, writing–review & editing. Fathollah Moztarzadeh: project administration, supervision.

## Conflicts of interest

There are no conflicts to declare.

## Supplementary Material
